# Isolation and Characterization of a Novel Jumbo Phage HPP-Temi Infecting *Pseudomonas aeruginosa* Pa9 and Increasing Host Sensitivity to Ciprofloxacin

**DOI:** 10.3390/antibiotics13111006

**Published:** 2024-10-25

**Authors:** Olufunke Olufunmilola Olorundare, Nikita Zrelovs, Dennis Kabantiyok, Karina Svanberga, Juris Jansons, Andris Kazaks, Godwin Ojonugwa Agada, Chibuzor Gerald Agu, Oluwatoyin Ruth Morenikeji, Ogundeji Alice Oluwapelumi, Thomas Dung, Shedrach Benjamin Pewan

**Affiliations:** 1 Department of Medical Microbiology, Clinical Sciences, University of Jos, Jos 930105, Nigeria; 2 Forest Research Institute of Nigeria, Federal College of Forestry, Jos 930105, Nigeria; 3 Latvian Biomedical Research and Study Centre, LV-1067 Riga, Latvia; nikita.zrelovs@biomed.lu.lv (N.Z.); karina.svanberga@biomed.lu.lv (K.S.); jansons@biomed.lu.lv (J.J.); andris@biomed.lu.lv (A.K.); 4 Fleming Sentinel Lab for AMR, National Veterinary Research Institute NVRI, Vom 930001, Nigeria; denzelkbt@gmail.com (D.K.); goaagada@gmail.com (G.O.A.); alice.ogundeji@nvri.gov.ng (O.A.O.); drthomasdauda@gmail.com (T.D.); 5 Bacteria Research National Veterinary Research Institute NVRI, Vom 930001, Nigeria; cagu@rocketmail.com; 6 West African Centre for Emerging Infectious Diseases, Jos University Teaching Hospital, Jos 930241, Nigeria; oluwatoyinmorenikeji28@gmail.com; 7 Extension Division, National Veterinary Research Institute NVRI, Vom 930001, Nigeria

**Keywords:** jumbo phage, *Pseudomonas* phage, *Pawinskivirus*, whole-genome sequencing, comparative genomics, phage-antibiotic synergy

## Abstract

*Pseudomonas aeruginosa* is a bacteria responsible for many hospital-acquired infections. Phages are promising alternatives for treating *P. aeruginosa* infections, which are often intrinsically resistant. The combination of phage and antibiotics in clearing bacterial infection holds promise due to increasing reports of enhanced effectiveness when both are used together. The aim of the study is to isolate and characterize a novel *P. aeruginosa* phage and determine its effectiveness in in vitro combination with antibiotics in controlling *P. aeruginosa*. In this study, a novel jumbo myophage HPP-Temi infecting *P. aeruginosa* Pa9 (PP334386) was isolated from household sewage. Electron micrographs of the phage were obtained to determine the morphological features of HPP-Temi virions. Complete genome analysis and a combination of Pseudomonas phage HPP-Temi with antibiotics were examined. The phage HPP-Temi was able to productively infect *P. aeruginosa* ATCC 9027 but was unable to infect a closely related genus. The phage was stable at 4–37 °C, 0.5% NaCl, and pH 8 for at least one hour. The HPP-Temi genome is a 302,719-bp-long dsDNA molecule with a GC content of 46.46%. The genome was predicted to have 436 ORFs and 7 tRNA genes. No virulence factor-related genes, antimicrobial resistance, or temperate lifestyle-associated genes were found in the phage HPP-Temi genome. Phage HPP-Temi is most closely related to the known or tentative representatives of the *Pawinskivirus* genus and can be proposed as a representative for the creation of a novel phage species in that genus. The phage and antibiotics (Ciprofloxacin) combination at varying phage titers (10^3^, 10^6^, 10^9^) were used against *P. aeruginosa* Pa9 (PP334386) at 3.0 × 10^8^ CFU/mL, which was carried out in triplicate. The result showed that combining antibiotics with phage significantly reduced the bacteria count at 10^3^ and 10^6^ titers, while no growth was observed at 10^9^ PFU/mL. This suggests that the effect of phage HPP-Temi in combination with antibiotics is a potential and promising agent for the control of *P. aeruginosa* infections.

## 1. Introduction

The golden era of antibiotic discovery, which lasted for about four decades, from 1928, when Flemming discovered Penicillin, to the 1960s, was interposed by the emergence of resistant species [1]. Through the years, microorganisms have learned how to resist antimicrobial therapy as a result of extensive use and abuse [2]. Antimicrobial resistance (AMR) has become a global phenomenon, with pandemic projections of about 10 million AMR-related deaths annually by 2050 [3]. Despite the need for improved treatment outcomes, little has been achieved due to the slow pace of discovering novel antimicrobials [4]. Although advances in modern drug discovery, such as genome mining, are creating pipelines for discovering novel bioactive compounds, researchers are yet to catch up with the emergence of drug-resistant bacterial strains or translate the successes to drugs on the counter. Following the slow pace of novel antimicrobials, the number of AMR-related deaths has risen steeply. In 2019, AMR-related deaths reached a concerning level of 1.2 million [3], with many of these cases reported in sub-Saharan Africa [5]. Solutions for combating antimicrobial resistance are currently an active area of research in infection biology, with research on bacteriophages and antimicrobial peptides in the spotlight.

Before the clinical application of antibiotics, prospects of bacteriophages (i.e., phages) as antibacterial agents were considered by several researchers due to their lytic effects on bacterial pathogens. Felix d’Herelle’s first use of phages to treat bacterial dysentery birthed the therapeutic use of phages. Researchers in Germany, the USA, and France were on the frontlines of commercial phage products [6]. This area of research coincided with the golden era of antibiotics, which became a drawback. Phage research was abandoned throughout most of the world due to the increase in novel antibiotics [7], their broader applications, and the prospects of antibiotics beyond the clinical environment. Recent concerns for options in treatment outcomes have necessitated renewed interest in phage [8,9].. Although bacterial cells can adapt or even evolve changes to resist phage lysis, these changes are often targeted at surface structures that serve as phage adsorption receptors [10,11]. While these changes are targeted at phage resistance and disrupting the phage cycle, it can be argued that these changes may simultaneously downplay the host’s virulence, making it more susceptible to immune or antimicrobial suppression. Phage advantages over traditional drug development methods, particularly antibiotics, lie in their target action and specificity to their bacterial host. Phages that target pathogenic bacteria without harming beneficial bacteria can be isolated and used. On the other hand, traditional antibiotics often have a broad spectrum of action, which can disrupt healthy bacteria populations as a collateral damage [12]. Phage therapy can reduce the development of antimicrobial resistance, and antibiotic resistance is a steadily growing global health crisis [13], and has faster development times and adaptability; phages are naturally occurring and can be readily isolated from the environment [14]. Phages generally have low toxicity to humans and are highly specific to host bacteria. Antibiotics, while safe for most people, can still cause reactions ranging from mild allergic responses to severe effects like kidney or liver damage.

Although several factors limit phage therapy, it has proven effective in countries like Russia, Poland, Finland, and Georgia, where its application remains in practice [15,16]. There has been documented evidence of phage therapy in clearing infections caused by multidrug resistant bacterial pathogens [17]. While subtherapeutic doses can be explored, the combination of full therapeutic doses of antibiotics with phages is more typical in clinical treatment [18]. Scientists from Eliava, Georgia, in the 1970s, conducted the first clinical trials to evaluate the efficacy of phage cocktail intravenous administration against an array of staphylococcal infections and discovered that 23% of those treated with antibacterial products recovered, and 41% treated with phages recovered; however, those treated with a combination of phage cocktail and antibiotics showed a higher recovery rate of 78%. Similar approaches to cystic fibrosis have produced fruitful outcomes [6]. While phage-antibiotic synergy (PAS) presents viable results in specific scenarios, it is still riddled with mixed outcomes. Using phage to enhance antibiotics or vice versa has produced dissimilar results, yet concurrent administration of both is effective in treating infections [19]. Consequently, PAS would be selected depending on the observed outcomes for the individual pathogen.

*P. aeruginosa* is a Gram-negative, motile, aerobic, and facultatively anaerobic bacterium that can populate various environments such as soil, water, plants, and animals [20]. *P. aeruginosa* is an opportunistic human pathogen that causes infections in wounds and respiratory and urinary tracts. It is one of the major causes of hospital-acquired infection, with the leading cause of morbidity and mortality rates [21,22]. It is responsible for 22% of hospital-acquired infections [23], and mortality rates associated with *P. aeruginosa* are related to multidrug-resistant isolates, particularly in persons with blood-borne infections (43.2–58.8%) [24] and underlying health conditions [25], particularly in cystic fibrosis patients. Mortality rates of up to 44.6% have been associated with MDR *P. aeruginosa* infection.

*P. aeruginosa* has been one of the poster pathogens in the ESKAPE (*Enterococcus faecium*, *Staphylococcus aureus*, *Klebsiella pneumoniae*, *Acinetobacter baumanii*, *P. aeruginosa*, and *Enterobacter* spp.) group, which are notorious for their roles in contributing to global antimicrobial resistance crisis [26]. It is also a known agent in nosocomial infections, urinary tract infections, cystic fibrosis patients, surgical wounds, and bloodstream infections [27,28,29]. Additionally, *P. aeruginosa’s* intrinsic features, such as particular porins, multidrug efflux pumps, and biofilm formation ability, foster its resistance to several antibiotics, including aminoglycosides, fluoroquinolones, beta-lactams, and polymyxins [30]. The use of bacteriophage in combating drug resistance in *P. aeruginosa* was first documented in 1957 by Kellenberger [31]. Since then, infection studies using animal models on the suitability of phages in combating AMR by *P. aeruginosa* have been documented [32]. Although the success of these studies is yet to be integrated into clinical settings, their successes will form the foundation upon which future phage therapy would benefit.

In this study, phage HPP-Temi was isolated from household sewage and characterized. The virion morphology of HPP-Temi was determined by transmission electron microscopy. The lytic ability and stability of bacteriophage HPP-Temi under different salinity, temperature, and pH were determined. The in vitro effect of bacteriophage HPP-Temi was demonstrated in combination therapy with antibiotics against *P. aeruginosa* Pa9 (PP334386). A complete genome sequence of phage HPP-Temi was obtained using Next-Generation Sequencing and functionally annotated and is publicly available under the following accession number: PP968062. Prospects for future applications were presented because of the significant contribution of *P. aeruginosa* to global AMR spread.

## 2. Results

### 2.1. Isolation of Bacteriophages

**Figure 1:**
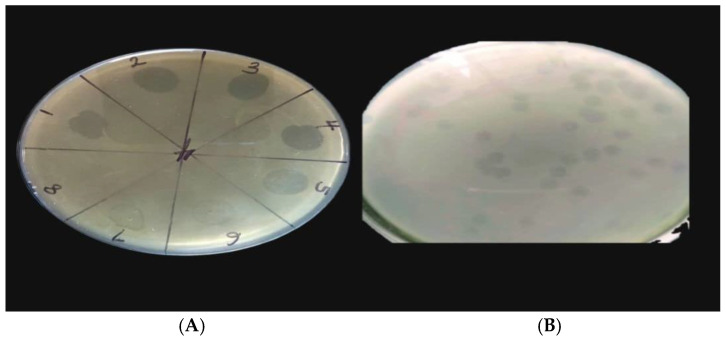
(**A**) (3.43 mm) is the zone of lysis of phage HPP-Temi on *P. aeruginosa* Pa9 lawn showing good lytic activity. (**B**) (3.65 mm) shows plaques of Phage HPP-Temi on the lawn of *P. aeruginosa* Pa9 at 37 °C.

The isolation of bacteriophages specific to the *P. aeruginosa* Pa9 isolate from household sewage samples was successful. *P. aeruginosa* Pa9 is a veterinary isolate with accession number PP334386. Phage HPP-Temi produced a large clear zone on the lawn culture of *P. aeruginosa* Pa9 (Figure 1A). Plaque morphology appears to be large, clear, and round (Figure 1B). Hence, distinct plaques were selected, purified, and stored for use.

### 2.2. Morphological Features

**Figure 2:**
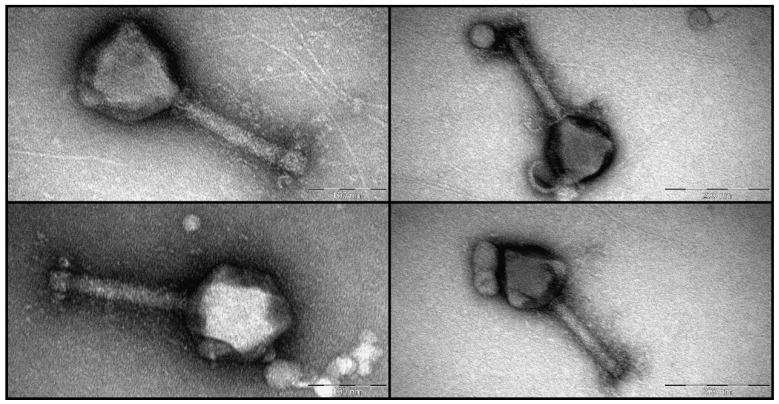
Collage of the transmission electron micrographs demonstrating seemingly intact virions of *Pseudomonas* phage HPP-Temi taken from crude lysate negatively stained with 0.5% uranyl acetate. Note the different magnifications for the right and left side micrographs (the scale bars correspond to 100 and 200 nm, respectively).

Intact virions of *Pseudomonas* bacteriophage HPP-Temi demonstrate characteristics of a myophage morphotype–icosahedral capsid with a long contractile tail attached. Phage HPP-Temi capsids have a diameter of approximately 128 ± 8 nm. The uncontracted HPP-Temi tail length (distance from the tail junction with the capsid to the distal base plate tip) is approximately 182 ± 6 nm with an uncontracted tail width of ~23 ± 1 nm (measured at the midpoint). From the micrographs, a relatively “lush” wide baseplate is discernable, albeit with a resolution precluding precise measurements thereof (Figure 2).

### 2.3. Phage Stability Under Different Chemical and Physical Conditions

**Figure 3:**
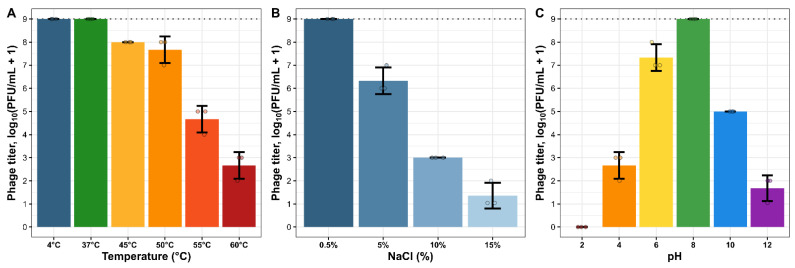
Stability of phage HPP-Temi virions in different physio-chemical conditions. (**A**) Stability under thermal treatment. (**B**) Stability in different NaCl concentrations. (**C**) Stability in different pH. In all the tiles, the initial HPP-Temi titer of the sample at the start of the experiment is indicated by the black dotted line (~10^9^ PFU/mL). Bar heights represent the mean, and error bars indicate ± one standard deviation. Jittered points represent individual measurements.

We observed that the phage titer decreases at higher temperatures, with the least titer recorded at 60 °C, with a reduction of 6 × log10, while the most stable titer values were observed at 4 °C and 37 °C and 45–50 °C. Although the phage between 45 and 50 °C showed a little reduction in titer of 1log 10, the phage titer witnessed a steep drop from temperatures above 50 °C with 4 × log10 (Figure 3A). 4 × log10 reduction was observed between pH 8 and pH 10. Phages were stable at a pH range of 8.0, while a decrease in titers of 2 × log 10 was observed between pH values of 8.0 and 6.0. At pH 2, 100% inactivation of the phage was observed (Figure 3C). Phage retained stability in the presence of 0.5% NaCl with no titer reduction (Figure 3B), while there was a great reduction in titer of more than 2 × log10 at 5%, a reduction of more than 4 × log between 10% and 15% NaCl.

### 2.4. Complete Genome Analysis

**Figure 4:**
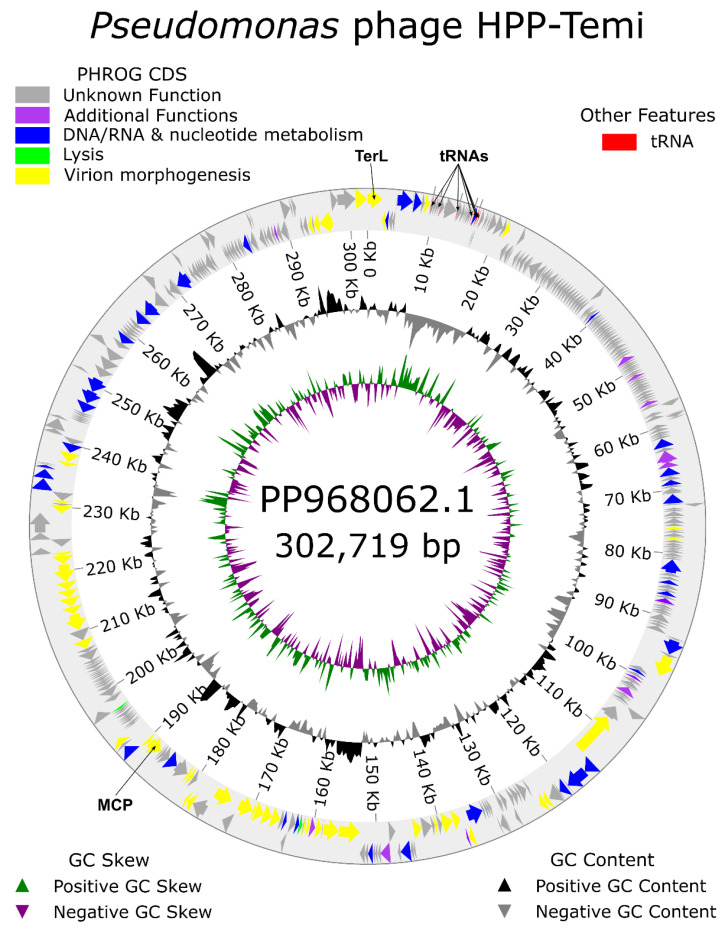
Genome organization of the phage HPP-Temi. Arrows representing open reading frames or tRNA genes point in the direction of the transcription and are color-coded based on the functional category of their putative product according to the legend. The outermost ring shows direct strand features, and the second outermost ring–complementary strand features. Selected features of interest are additionally annotated with labels. The innermost ring shows GC skew and the second innermost ring shows GC content.

The complete genome of *Pseudomonas* bacteriophage HPP-Temi is a 302,719 bp long dsDNA molecule with a GC% of 46.46. Within this genome, 436 putative protein-coding open reading frames (ORFs) were predicted, as well as seven transport RNA genes. Functional annotation of putative HPP-Temi ORF products allowed us to predict functions for less than 33% of presumed proteins, with the vast majority (336 predicted proteins to be exact) currently remaining without any functional annotation (Figure 4).

The complete annotated genome sequence of *Pseudomonas* phage HPP-Temi is publicly available from GenBank under the accession number PP968062.

### 2.5. Genomic Similarity to Other Phages

BLASTN search against the publicly available *Caudoviricetes* phage genomes using the complete genome sequence of *Pseudomonas* phage HPP-Temi has revealed four closely related phages, alignments to which gave 92–93% query coverage with an identity of 80–81% (to phages PC1C, BRkr, vB_PaeM_PS119XW, and vB_PaeM_phiLGB22), which were followed by hits resulting in more distant alignments (e.g., 45% query coverage of ~74% identity for PhiPA3, and multiple hits with query coverages of around 30%).

**Figure 5:**
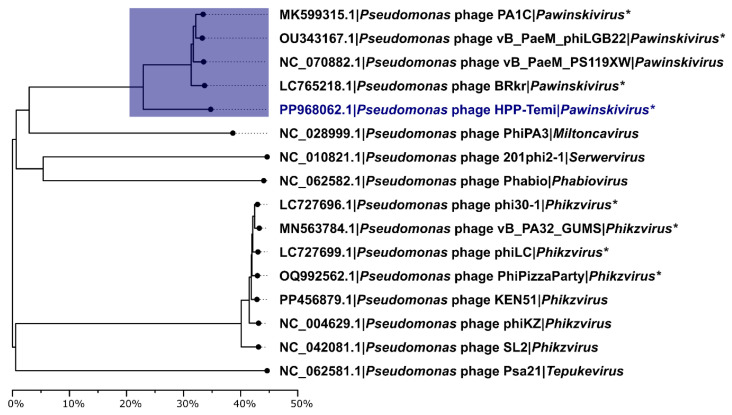
Intergenomic distance neighbor-joining tree of the isolated *Pseudomonas* phage HPP-Temi and its relatives from the public biological sequence repositories. The clade comprising HPP-Temi and other recognized or proposed *Pawinskivirus* phage genus representatives is highlighted by the blue rectangle. The label of the tip corresponding to phage HPP-Temi is colored blue. The tree is drawn to scale and is in units of VIRIDIC-calculated intergenomic distance percentages. Tip labels are in the form of “Genome accession number | Phage name | Genus”. An asterisk (*) after the genus name indicates that a given phage is not yet a part of the official phage taxonomy.

The genomes corresponding to these phages were downloaded and analyzed using VIRIDIC, also using several more distinct RefSeq sequence hits (at least 20% query coverage). The latest ICTV-ratified taxonomy was used to extrapolate genus and species-level designations among the phages found within the dataset, where relevant, and the intergenomic distance neighbor-joining tree, including multiple recognized *Pseudomonas* jumbophage genera representatives related to HPP-Temi, was drawn. The clustering pattern suggested that HPP-Temi, indeed, shows genus-level similarity to several other jumbophages from the *Pawinskivirus* genus and has a way more distant relation to either recognized or tentative *Miltoncavirus*, *Serwervirus*, *Phaviovirus*, as well as *Phikzvirus* and *Tepukevirus* phage genera representatives (Figure 5).

To compare shared gene contents of the dataset comprising vB_PaeM_PS119XW, HPP-Temi, and other tentative representatives of the *Pawinskivirus* genus, the initial annotation differences posed by likely different genome annotation approaches utilized by the original sequence annotators were removed by reannotating all the downloaded genomes in a standardized manner (e.g., no genomic feature annotations were seen in the GenBank sequence of vB_PaeM_phiLBG22 at all, Table 1). After reannotation, all the tentative *Pawisnkivirus* representatives isolated at presumably different times and from different sources (at least speaking of the expected geographical origin of the samples) had not only comparable genome sizes of 301,543–306,291 Kbp but also comparable predicted CDS counts (424–447) and tRNA gene number (7–8). Notably, the GC% content of the HPP-Temi genome was nearly 3% higher than that of other phage isolates, tentatively representing the same other species within the *Pawinskivirus* genus.

**Figure 6:**
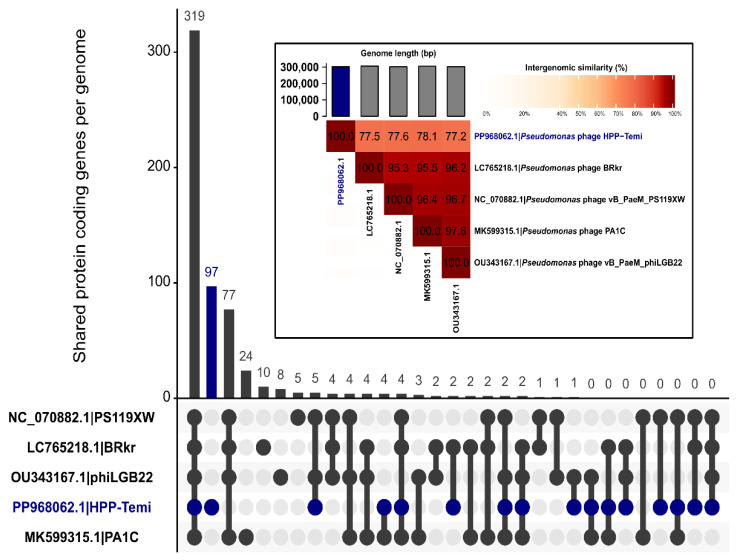
Similarities between *Pawinskivirus* representatives. Shared protein-coding gene counts across genomes are shown for either all the phages analyzed, the intersections of particular different phages, or individual phages. Only genes encoding for proteins having an identity of more than 70% are considered to be shared. An inset shows a heatmap demonstrating pairwise intergenomic similarity percentages between the same bacteriophages.

The pangenome of all of these five tentative *Pawinskivirus* isolates comprised 583 genes, of which 319 or up to more than 71% of the genes were shared, with tentative *Pawinskivirus PS119XW* isolates also having 77 more shared genes in common among themselves. HPP-Temi had 97 of the genes whose products were less than 70% similar to any of the proteins found in other phages within the analysis (Figure 6).

### 2.6. Phage-Antibiotics Combination Against P. aeruginosa

**Figure 7:**
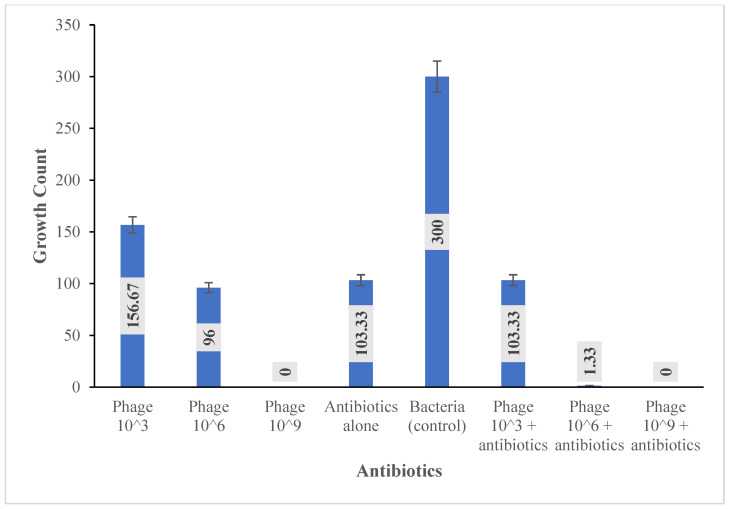
Phage antibiotic combination effect on *P. aeruginosa.* The statistical analysis was determined by a two-way ANOVA and means separated according to the Duncan multiple range test (Supplementary 2), which showed a significant reduction in bacterial growth. Phage 10^9^ and phage 10^9^ + antibiotics showed no bacteria growth.

The result of the combination of antibiotics and phage HPP-Temi (10^3^, 10^6^, and 10^9^ PFU/mL) showed inhibitory effects at varying concentrations (Figure 7). Ciprofloxacin (5 μg) was selected as one of the approved antibiotics for the treatment of *P. aeruginosa* infection according to performance standards for antimicrobial susceptibility testing. This was used for the combination effect by determining the CFU/mL after overnight incubation at 37 °C. *P. aeruginosa* Pa9 at 3.0 × 10^8^ CFU/mL was used as a control (without phage or antibiotics). The phage and antibiotic combination at varying phage titers (10^3^, 10^6^, 10^9^ PFU/mL) showed a synergistic effect by reducing the bacterial count at 10^3^ to 1.0 × 10^2^ CFU/mL, and at 10^6^, only scanty growth was observed. No bacterial growth was observed at 10^9^ PFU/mL in combination with antibiotics after overnight incubation. When compared with antibiotics alone, phages at 10^3^ PFU/mL in combination with antibiotics showed no significant difference, whereas phages with 10^6^ and 10^9^ PFU/mL in combination with antibiotics showed a significant difference. Phage combination with antibiotic at 10^3^, 10^6^, and 10^9^ PFU/mL with control isolate at 3.0 × 10^8^ shows a significant difference (*p* < 0.05).

## 3. Discussion

The imminent threat of antimicrobial resistance to global health has sparked renewed interest in Phage research as an alternative to antibiotics [33]. *P. aeruginosa* is a common nosocomial pathogen that causes life-threatening infections in humans, and to overcome the antibiotic resistance of pseudomonal infections [34], we investigated phage therapy as an alternative. We isolated Phage HPP-Temi, a *P. aeruginosa* Pa9 virion isolated from household sewage around Jos, Plateau State, Nigeria. The host range of the phage was examined against *P. aeruginosa* ATCC 9057 obtained from the Fleming Laboratory NVRI Vom, which showed lytic activity, and closely related genera such as *E. coli* ATCC 13883, *Salmonella typhimurium* ATCC14028, and *S. enteritidis* 1145s with no lytic activity. Electron micrographs of the phages were obtained to determine the morphological features of HPP-Temi virions.

### 3.1. Virions of Pseudomonas Phage HPP-Temi

The relatively large capsid size of HPP-Temi, greatly exceeding in volume those of most myophages, is not unexpected due to it having to accommodate a long dsDNA genome of more than 300 kbp. The virion dimensions of HPP-Temi, however, resemble those of the other jumbo phages and roughly correspond to the early TEM-based phage PhiKZ, which is considered “the first of giants”, virion dimension measurements [35].

Environmental factors play important roles in the viability of phages. Phage stability characteristics reveal that temperature affects the infectivity of phage and an increase in the incubation temperature decreases phage titers, particularly at temperatures above 60 °C. It was evident that 4 °C and 37 °C are temperatures at which HPP-Temi retains infectivity. At 45–55 °C, the phage had just a little reduction in titer, and there was a high drop in titers at temperatures of 60 °C. Exposure to temperatures of 60 °C damages virions of HPP-Temi resulting in a great reduction in phage titers (Figure 3A). Overall results showed that the stability of these phages is negatively affected by increased exposure to high temperatures. pH is an important factor influencing the attachment, replication, and multiplication of phages. The effect of the pH of the medium on the stability of phage was determined by suspending the phage in pH-adjusted SM buffer, and the reduction in phage titer was determined. Phage particles showed great stability at pH 8, while a decrease in titers was observed at lower pH values of 6.0 and below and higher values above 8.0. At pH 2, 100% inactivation of the phage was observed (Figure 3C). This suggests that pH can interfere with phage virion structural integrity, preventing attachment to receptor sites on the host. This agrees with Abdelsattar et al. [36] and Sun et al. [37], who reported a decrease in titer at low and high pH. Phage retained stability in the presence of 0.5% NaCl (Figure 3B) with no titer reduction. A reduction in titer was observed in the presence of 5% NaCl and a further reduction in titer at 10% and 15% NaCl. High salt concentrations probably also affect the virion structural stability and denature the proteins of phages. This research was reported and demonstrated by Sharma et al. [38] that after 18 h of incubation under different pH conditions, phage DRL-P1 was most stable at pH 6.0, 7.0, and 8.0, and no significant loss in the titer was observed. However, below pH 3.0 and above pH 10.0, there was a decrease in phage titer as compared to the initial titer, and no viable phage was recovered after incubation at pH 1.0, 2.0, 13.0, and 14.0.

Sequencing of the genomic DNA and genome analysis help ascertain if phage could be suitable for phage therapy and does not harbor any genes that encode for antimicrobial resistance and/or toxin/virulence genes [39]. Some concerns regarding using phages with harmful genes can be solved by complete genomic characterization of the phages with genome sequencing, annotation of the genome, and determining the functions of genes. The knowledge of the functions of the genes greatly help to determine the suitability of phage for therapy [40].

### 3.2. The Complete Genome of Pseudomonas Phage HPP-Temi

Out of the ORF products with a function predicted, half could be categorized to be involved in virion morphogenesis (e.g., packaging, capsid, and tail assembly), although further studies would be necessary to conclude how many out of 50 such proteins are actually found within the mature infective HPP-Temi virions and which are playing a role only during the progeny virion maturation within the host. However, the sheer size of the HPP-Temi genome and the number of its genomic features allow us to expect a great deal of regulatory complexity. This is further supported by an observation that ORFs categorized in independent functional groups are interspersed with each other and relatively frequent coding strand shifts are seen. The expected boundaries for functionally related gene modules/operons are not evident from the annotated genome map at all (Figure 4).

No genes implicated in lysogeny or production of known virulence factors or antibiotic resistance genes could be identified, indicating that phage HPP-Temi might be considered a seemingly genomically safe candidate for further elaborate investigations of the possibility of its deliberate application to control *P. aeruginosa*.

Despite an average sequencing depth of 126× for bases of the HPP-Temi genome and appropriately prepared NGS libraries (random physical shearing for fragmentation and not a transposase-based library prep), no defined genome molecule termini could be identified using PhageTerm. Furthermore, TerL phylogeny reconstruction-based analysis within the context of bacteriophages with verified genome termini types/packaging strategies suggests that the so-called “headful” genome packing strategy is likely to be employed by HPP-Temi (Figure S1). This means that it is expected that HPP-Temi progeny phages package more than a single genome length into their procapsids, likely resulting in the virions of the HPP-Temi progeny population having redundant and circularly permuted genomes among them. As is common in such cases, the “(pseudo) circular” assembly representing the complete genome of HPP-Temi was “opened” at the predicted terminase large subunit encoding ORF start codon.

The complete annotated genome of *Pseudomonas* bacteriophage HPP-Temi is publicly available from GenBank under the accession number PP968062.1.

### 3.3. Genomic Similarity to Other Phages

The analysis demonstrated that HPP-Temi clusters together with isolate vB_PaeM_PS119XW, representing phage species *Pawinskivirus PS119XW*, the sole recognized representative of the phage genus *Pawinskivirus* to date, at the intergenomic distance, which allows us to consider HPP-Temi as a representative of the same genus as well. Interestingly, the pairwise intergenomic similarities among the top 4 BLASTN hits to HPP-Temi allow us to consider all these phages as different isolates of the same *Pawinskivirus PS119XW* (intergenomic similarities of more than 95% across the complete genome lengths). Essentially, this indicates that HPP-Temi should be proposed as an isolate for the creation of the second phage species within the genus *Pawinskivirus*, as its intergenomic similarity to either of the isolates belonging to the *Pawinskivirus PS119XW* species lies greatly below the 95% species demarcation but below the genus demarcation threshold of 70% (Figure 6).

Although not immediately relevant to HPP-Temi, the results of this analysis related to *Pawinskivirus PS119XW* further raise the question regarding the mechanistic ICTV BVS phage genus and species-level intergenomic similarity demarcation criteria applicability when considering bacteriophages with larger genomes. Even the bacteriophage isolates tentatively belonging to this same species seem to have a notable amount of ORF products, which show pairwise similarity of less than 70%. Given paucity of comparable in-depth microbiological characterization data for multiple jumbo phage isolates seemingly representing the same species, one may only guess whether the presence of these 5–24 “unique” genes found in either tentative isolates of *Pawinskivirus PS119XW* results in phenotypic manifestations that might push for reconsideration of the currently widely-adopted intergenomic distance “go-to”s by taking the genome lengths of the bacteriophage groups they are applied to into account.

### 3.4. HPP-Temi Improves Ciprofloxacin Efficacy

The combination of antibiotics and phage for treating bacterial infections has been observed to improve sensitivity of *P. aeruginosa* in biofilms [34]. The result of the combination of antibiotics (Ciprofloxacin) and phage HPP-Temi (10^3^, 10^6^, and 10^9^ PFU/mL) had inhibitory effects (Figure 7). The effects of phages at 10^3^ PFU/mL in combination with antibiotics, ciprofloxacin, showed a reduction in the number of cells (7.3 × 10^1^ CFU/mL). A further reduction was at a higher phage titer at 10^6^ PFU/mL in combination with ciprofloxacin with no significant growth. Phages at a higher titer of 10^9^ PFU/mL with ciprofloxacin resulted in the complete elimination of bacterial cells. Even though similar effects were seen in the phage-only treatment, we believe that the clearing observed at 10^9^ PFU/mL combinations with Ciprofloxacin is solely due to Phage Temi. However, the phage-ciprofloxacin combination at titer 10^6^ showed significantly greater reduction when compared to phage-only treatment. We propose that at suboptimal concentrations, Phage Temi may have increased *P. aeruginosa*’s sensitivity to the antibiotic. Phage Temi’s ciprofloxacin synergistic effect in the elimination of *P. aeruginosa* is similar to the report of Chang et al. [41], who showed that the phage-ciprofloxacin combination has a better effect compared to the single-compound treatment. This is also corroborated by Manohar et al. [34], who reported phage Motto acting synergistically with all tested antibiotics and was effective at disrupting *P. aeruginosa* biofilms. The effect of *Pseudomonas* phage HPP-Temi and the concentration of the antibiotics on *P. aeruginosa* was determined by CFU/mL after overnight incubation, and this shows an interaction between phage and antibiotics. The phages with a titer of 10^3^ and 10^6^ PFU/mL notably reduced the bacterial growth to 103 and 133 CFU/mL, respectively. No bacterial growth was observed at a phage titer of 10^9^ PFU/mL. This shows that the phage increases antibiotic efficacy in a dose-dependent manner, such that the greater the phage concentration, the higher the antimicrobial effect it produces in combination with antibiotics. This is not surprising because it has been reported that MDR *P. aeruginosa*, when treated with phages, expresses restored antimicrobial susceptibility, triggered by evolutionary trade-offs due to loss of overexpression of efflux pumps [42]. Other studies support the phage-mediated sensitization effect on *P. aeruginosa* when combined with traditional antibiotics such as Streptomycin, Nalidixic acid, Gentamicin, and Polymyxin B. These combinations produce effects in varying degrees and, in some instances, total elimination of bacteria at higher phage titers [43,44]. While we admit that determining synergism using Fractional Inhibitory Concentration (FIC) is the gold standard, we believe the combined effect of phage versus antibiotics sets the stage for synergistic research. Studies on jumbo phages such as MIJ3 phiPA3, PA5oct, phiKZ, SL2, KTN4, PA02, PA7, and PaBG on clinical isolates of *P. aeruginosa*, show their potential as agents with a wide range of antimicrobial activity [45]. The combination effect produced after combining Ciprofloxacin and HPP-Temi could be promising in the treatment of *Pseudomonas* infection. However, the mechanism of action and interaction can further be investigated.

## 4. Materials and Methods

### 4.1. Bacterial Culture and Preparations and Sample Collection

*P. aeruginosa Pa9* strain (PP334386), which is a veterinary isolate, was used as a host for the isolation and characterization of the phage. The working stock was stored at 4 °C in Muller Hilton broth. For each experiment, the bacteria were grown overnight in 5 mL broth at 37 °C with 100 rpm agitation. A household Sewage sample was collected in the Jos North Local Government area of Plateau State, Nigeria, and screened as a source of bacteriophage. The sample was centrifuged at 10,000× *g* for 20 min to separate bacterial cells and debris. The supernatant was filtered through 0.45 µm filters, transferred to a clean tube, and stored at 4 °C [46].

### 4.2. Enrichment and Isolation of Bacteriophages

The isolation and enrichment of phages were done based on a method described by Ateba and Akindolire [47] with modifications. Briefly, the clarified sample (10 mL) was added to a 10 mL double-strength brain heart infusion with CaCl_2,_ and a 100 µL overnight culture of *P. aeruginosa* host strain was added. The suspension was incubated for 48 h with gentle shaking at 50 rpm at 37 °C. After incubation, the mixture was incubated in a shaker at 50 rpm for 48 h at 37 °C. After incubation, it was centrifuged at 5400× *g* for 15 min at 4 °C, and supernatants were filtered through 0.45 µm syringe filters. The filtrates were stored at 4 °C.

### 4.3. Preparation of Bacterial Lawn and Plaque Formation

The plaque formation and phage activity against *P. aeruginosa* were determined by the spot-test technique [47,48]. Then, 100 µL of an overnight broth culture of the *P. aeruginosa* was added to 3 mL of molten top agar (0.6% *w*/*v* agar) in a test tube at 50 °C and immediately poured onto Nutrient agar (NA) plates and allowed to solidify for 15 min. Plaque formation was determined by spotting 10 µL of stored filtrate (lysate) on the solidified top agar with the bacterial lawn and allowed to adsorb for about 10 min. After absorption, it was incubated at 37 °C overnight, and plates were observed for clear zones or zones of inhibition on bacterial lawns at the spot sites. The cleared zone was harvested from the top agar into 300 µL of Sodium Magnesium (SM) buffer in an Eppendorf tube using a pipette tip. The phage particles were allowed to diffuse by storing overnight at room temperature and then filtered through 0.45 µm syringe filters.

### 4.4. Phage Titration and Purification

A 1:10 dilution of isolated phage was made, according to the method [36], and phage was purified by adding 100 µL of overnight host bacteria culture at OD600 to tubes containing 10 µL of phage lysate dilution (10-5, 10-6, 10-7, 10-8 and 10-9) and incubated at 37 °C for 10 min. 3 mL of molten top agar was added, mixed, and poured on an agar plate. It was allowed to cool until the top agar solidified and then incubated overnight at 37 °C. Visible plaques were counted, and PFU/mL was determined [45]. Plaques were picked from the overlays and transferred into 300 µL SM buffer (buffer: 5.8 g/L, MgSO_4_. 7H_2_O, NaCl 2 g/L, 10 mL/L 1M Tris-HCl (pH 7.0)) and allowed to diffuse by incubating overnight at 4 °C. Phages were purified three times to obtain single plaques. Purified phage was serially diluted, and the phage titer was determined using the spot test technique. The purified phage lysate was stored at 4 °C.

### 4.5. Host Range Determination

*P. aeruginosa ATCC 9027* was used in the study [49]. The spot test was performed on a bacterial overlay. A drop of 10 µL of phage suspension 10^8^ PFU/mL was dropped on the bacterial lawn. The plate was incubated overnight at 37 °C and the lysis zone was observed. Closely related genera such as *E. coli* ATCC 13883, *Salmonella typhimurium* ATCC14028, and *S. enteritidis* 1145s were also tested for the zone of lysis.

### 4.6. Transmission Electron Microscopy

The TEM visualization of the bacteriophage particles (as well as their virion feature dimension measurements) was performed from the HPP-Temi-containing lysate negatively stained using 0.5% uranyl acetate as described in [50]. However, crude lysates rather than purified samples were used.

Briefly, virions were adsorbed onto the carbonized formvar-coated 300 mesh copper grids (Agar Scientific, Stansted, UK), rinsed with 1 mM EDTA, stained with 0.5% uranyl acetate solution, and air-dried before being analyzed through a JEM-1230 electron microscope (JEOL, Akishima, Japan). Bacteriophage HPP-Temi virion feature dimensions were measured using ImageJ (v1.52a; [51]) utilities guided by micrograph scale bars for pixel-to-nm conversion.

### 4.7. Thermal, pH, and Salinity Stability

The effects of environmental and chemical factors on the isolated phage, such as temperature, pH, and salinity, were determined [37,46]. The thermal stability of phage (10^9^ PFU/mL) was ascertained at different temperatures (4, 37, 45, 50, 55, and 60 °C) after 1 h of incubation. The effect of pH condition on phage (10^9^ PFU/mL) stability was determined at different pH levels (2, 4, 6, 8, 10, 12) by the addition of either 1M HCL or 1M NaOH and incubated at 37 °C for 1 h. The effect of different saline concentrations (0.05%, 0.5%, 10%, 15% NaCl) on the stability of the phage (10^9^ PFU/mL) was determined and incubated at 37 °C for 18 h. Phage diluted in SM Buffer was used as a control in all the experiments. Each treatment was performed in triplicate, and the phage titer was determined using the spot test technique.

### 4.8. Phage DNA Extraction

Phage HPP-Temi genomic DNA was extracted using the Zymo Inqaba Biotec DNA extraction kit. The cell was centrifuged and suspended at 5000× *g* for 5 min, and the supernatant was removed. 500 µL of genomic lysis buffer was added to pellet the cell. It was vortexed for 4–6 s and allowed to stand for 5–10 min at room temperature. A zymo-spin IICR column was transferred in a collection tube and centrifuged at 10,000× *g* for one minute and then transferred to a new collection tube. 200 µL of DNA pre-washed buffer was added to the spin column and centrifuged at 10,000× *g* for one minute. The spin column was transferred to a clean tube, and 50 µL DNA elution buffer was added; it was incubated for 2–5 min at room temperature and was then centrifuged.

### 4.9. Whole-Genome Sequencing

Approximately 200 ng of the dsDNA isolated from the HPP-Temi phage sample was randomly sheared using a Covaris S220 focused ultrasonicator (Covaris, Woburn, MA, USA) protocol designated for an average fragment length of 550 bp. TruSeq DNA nano low-throughput library prep kit (Illumina, San Diego, CA, USA) manufacturer’s instructions were followed to prepare the NGS library. Adapter #10 from TruSeq DNA single indexes set B (Illumina) was used to allow distinguishing HPP-Temi reads from those originating from other libraries sequenced in the same run. Quality and quantity of the resultant library were checked using an Agilent 2100 bioanalyzer (Agilent, Santa Clara, CA, USA) with a High Sensitivity DNA Kit (Agilent), as well as a Qubit fluorometer (Invitrogen, Waltham, MA, USA) dsDNA high-sensitivity quantification assay (Invitrogen). Finally, the library was pooled with other uniquely barcoded libraries and sequenced on the Illumina MiSeq system (Illumina) with the 500-cycle MiSeq reagent kit v2 nano (Illumina).

### 4.10. De Novo Assembly and Functional Annotation of HPP-Temi Genome

The read dataset corresponding to the HPP-Temi NGS library was evaluated using FastQC (v0.11.9; [51]) and then trimmed using the bbduk tool (v38.69, part of the bbmap package [52]). Possible adapter remainders that slipped through the demultiplexing as well as the trailing base of each read were trimmed off. Any reads that were shorter than 50 bp in length after the trimming procedure were discarded. The processed read dataset was forwarded as input for the de novo assembly using Unicycler (v0.4.8; [53]) in the “normal” mode.

Non-processed reads were used alongside the assembled (pseudo)circular contig representing the genome of phage HPP-Temi to predict the genome packaging strategy/termini type the phage utilizes with the help of PhageTerm (v1.0.12; [54]). Also, raw reads were mapped to the de novo assembled genome using BWA-MEM (v0.7.17-r1188; [55]) to calculate the mean sequencing depth.

Pharokka (v1.7.1; [56]) was the tool of choice for the functional auto-annotation of the genome. PHANOTATE was chosen for the ORF calling, while both ARAGORN [57] and tRNA scan [58] were chosen for tRNA gene calling. Antibiotic resistance gene or virulence factor presence in the predicted proteome of HPP-Temi was assessed by comparisons of tentative HPP-Temi protein amino acid sequences to CARD [59] and VFDB [60] databases, respectively. Due to the size and complexity of the HPP-Temi genome, no manual annotation curation was attempted.

The genome organization plot was generated using the “pharokka_plotter.py” script running the pyCirclize Python package under the hood [56,61] and touched up in Inkscape (v. 1.0.1; available online: https://inkscape.org (accessed on 10 May 2021).

The complete auto-annotated genome sequence of the *Pseudomonas* phage HPP-Temi was deposited to GenBank and was assigned the following accession number: PP968062.1.

### 4.11. Determination of Relationship to Other Sequenced Phages

The complete genome nucleotide sequence of phage HPP-Temi was queried against the sequences originating from phage order *Caudoviricetes* representatives publicly available (taxid: 2731619) using the BLASTN [62] web server, and the top 10 hits with the highest total score were downloaded. Complete genomes of RefSeq hits having at least 20% query coverage that were not among the top ten hits were downloaded as well. VIRIDIC (v1.0; [63]) analysis was carried out under default settings to calculate the pairwise intergenomic distance matrix. This matrix was then used to create and export a neighbor-joining tree using the package “ape” (v. 5.5; [64]) capabilities in R. The resulting tree was visualized, midpoint rooted, and annotated using FigTree (Rambaut, A. FigTree v. 1.4.4; available online: http://tree.bio.ed.ac.uk/software/figtree/ (accessed on 10 May 2021)) and Inkscape (v. 1.0.1; available online: https://inkscape.org (accessed on 10 May 2021)).

### 4.12. Shared Gene Content Analysis with the Most Closely Related Phages

Genome nucleotide sequences of bacteriophages showing intergenomic distance of less than 30% to that of HPP-Temi were downloaded from GenBank (accessioned under NC_070882.1, LC765218.1, MK599315.1, OU343167.1) and reannotated as described in the third paragraph of the section “De novo assembly and functional annotation of HPP-Temi genome” to ensure functional annotation consistency for pangenome analysis.

Roary pangenome analysis pipeline (v. 3.13.0; [65]) was used under the minimum percentage identity threshold of 70% for BLASTP on Pharokka-generated general feature format (*.gff) files with the results of the autoannotation of HPP-Temi and its closest relatives.

Gene presence-absence table was imported into R, and UpSetR package (v. 1.4.0; [66]) capabilities were used to visualize shared protein-coding gene numbers among the phages of interest.

The intergenomic similarity matrix was calculated and visualized with the help of the VIRIDIC Web version [67].

The relevant figures were manually touched up and combined in Inkscape (v. 1.0.1; available online: https://inkscape.org (accessed on 10 May 2021)).

### 4.13. Genome Packing Strategy Prediction for Proposed Pawisnkivirus Representatives

The terminase protein amino acid sequences of phage HPP-Temi, as well as other phages showing genus-level similarity to it, were subject to the prediction of the packaging strategy they use by the merit of a TerL aa sequence phylogeny reconstruction with an extended dataset previously compiled by Merrill and colleagues [39], which comprises terminase/TerL sequences from bacteriophages whose genome molecule physical termini/packaging strategies were experimentally validated.

Sequences were aligned using MAFFT (v. 7.525; [68]), and the multiple sequence alignment was imported into MEGA (v. 7.0.26; [69]), where the neighbor-joining [70] tree was generated assuming uniform rates among sites from the MSA columns that had more than 90% site coverage, using substitutions per site as a measure of evolutionary distances and 1000 bootstrap [71] replicates to assess branch supports. The resulting tree was visualized and annotated using FigTree (Rambaut, A. FigTree v. 1.4.4; available online: http://tree.bio.ed.ac.uk/software/figtree/ (accessed on 10 May 2021)) and Inkscape (v. 1.0.1; available online: https://inkscape.org (accessed on 10 May 2021)).

### 4.14. Phage Antibiotics Combination

The phage antibiotic combination against *P. aeruginosa* to determine the synergistic effect was conducted using the broth tube dilution method by Manohar et al. [34] and Abdelsattar et al. [36] with modification. The phage was used at concentrations of 10^9^, 10^6^, and 10^3^ PFU/mL. Antibiotics 500 mg/mL were diluted to obtain a concentration of 5 µg according to CLSI [72] for ciprofloxacin and mixed with 50 µL of phage HPP-Temi at respective concentrations (10^9^, 10^6^, and 10^3^ PFU/mL) in microtiter plates containing nutrient broth, and 5 µL of *P. aeruginosa* Pa9 at 3 × 10^8^ CFU/mL were added and incubated overnight at 37 °C. After incubation, 100 µL was serially diluted up to 10^−6^ and plated on nutrient agar plates incubated overnight. CFU/mL was determined and compared with the control (Phage and antibiotics alone). The experiment was carried out in triplicate.

## 5. Conclusions

The study isolated a novel lytic jumbo myophage HPP-Temi, showing good lytic effectiveness against its isolation host *P. aeruginosa* Pa9 and *P. aeruginosa* ATCC 9027. The stability of HPP-Temi virions under various therapeutically relevant environmental conditions, coupled with its genome harboring no known virulence factors or antibiotic resistance genes, supports its safety as a putative candidate for possible therapeutic applications. Within the context of the uncovered bacteriophage diversity, *Pseudomonas* phage HPP-Temi can be proposed as an isolate that might serve to create a novel species in the phage genus *Pawinskivirus*. Notably, the observed effect of combining HPP-Temi with ciprofloxacin demonstrates enhanced bacterial growth inhibition than either of the agents alone, particularly at higher phage titers. This finding underscores the untapped potential of phage-antibiotic combinations (especially those with jumbo phages, which are not frequently isolated) in combating antibiotic-resistant infections. Further research, including in vivo studies and expanded host range testing on epidemiologically relevant strains, will be crucial in the follow-up investigation of the viability of phage HPP-Temi as a possible alternative or additive in the treatment of *P. aeruginosa* infections. These results contribute to the growing body of evidence supporting the use of bacteriophages and antibiotics as effective agents against multidrug-resistant pathogens.

## 6. Limitations

The study illustrates the combined effect of phage HPP-Temi and ciprofloxacin on the *P. aeruginosa* Pa9 cells when combined. The authors admit that determining synergistic effect through fractional inhibitory concentration (FIC) was not conducted, as the data presented are suggestive of improved efficacy and potential synergistic benefit. Furthermore, we could not work with other isolate strains to determine the breadth of the host range for HPP-Temi, synergy with other antibiotics was also not tested, and PFU/mL of phage was not determined after the synergy experiment.

## Figures and Tables

**Table 1:** General properties of the *Pseudomonas* phage HPP-Temi and other officially recognized (vB_PaeM_PS199XW) or proposed (others) *Pawinskivirus* genus representatives.

Accession	Phage	Genome Length (bp)	GC Content (%)	Submission CDS Count	Reannotated CDS Count	Hypothetical Proteins (%)	tRNAs	Species	Submitting Country	Collection Date	Isolation Source
PP968062.1	*Pseudomonas* phage HPP-Temi	302,719	46.46%	436	436	77.06%	7	Proposed as sp. nov.	Nigeria	25 September 2023	Household sewage
NC_070882.1	*Pseudomonas* phage vB_PaeM_PS119XW	301,543	43.62%	389	424	77.83%	7	*Pawinskivirus PS119XW*	Poland	Unknown	unknown
LC765218.1	*Pseudomonas* phage BRkr	306,291	43.47%	391	429	77.62%	7	Pawinskivirus PS119XW *	Japan	21 July 2022	Wastewater
MK599315.1	*Pseudomonas* phage PA1C	304,671	43.63%	401	447	77.63%	8	Pawinskivirus PS119XW *	Russia	Unknown	unknown
OU343167.1	*Pseudomonas* phage vB_PaeM_phiLBG22	302,555	43.60%	0	432	77.78%	7	Pawinskivirus PS119XW *	France	Unknown	unknown

* Indicates the proposed categorization.

## Data Availability

Data are contained within the article and Supplementary Materials.

## Supplementary Materials

The following supporting information can be downloaded at: https://www.mdpi.com/article/10.3390/antibiotics13111006/s1, Figure S1. Neighbor-joining tree of terminase/terminase large subunit (TerL) amino acid sequences for the prediction of the tentative genome packaging strategy *Pawinskivirus* representatives use. Tip labels are colored based on the distinct packaging strategies the phages seen in the tree use (experimentally verified for most of the phages represented in the tree [39]). The fraction of replicate trees in which the associated sequences clustered together in the bootstrap test (1000 replicates) is shown next above the branches for branches having bootstrap support higher than 0.8 (such branches also have their distal nodes colored in green). The tree is drawn to scale, and branch lengths represent the number of amino acid substitutions per site. Tip labels correspond to the phages from which the respective terminase/TerL amino acid sequences were derived and are in the format of “Protein accession|Phage”. Colored bars next to the labels indicate evolutionary distinct TerL clades, which correspond to different packaging strategies (LDTR stands for long direct terminal repeats, SDTR—short direct terminal repeats). The analysis involved 50 amino acid sequences. All multiple sequence alignment positions with less than 90% site coverage were eliminated. That is, fewer than 10% alignment gaps, missing data, and ambiguous bases were allowed at any position. There was a total of 267 positions in the final data set used to generate the tree. Supplementary 2.
