# Supplementary material for: Isolation and Characterization of a Novel Jumbo Phage HPP-Temi Infecting Pseudomonas aeruginosa Pa9 and Increasing Host Sensitivity to Ciprofloxacin
Source: Antibiotics (Basel). 2024 Oct 25;13(11):1006. doi: 10.3390/antibiotics13111006 (PMC11591403; doi:10.3390/antibiotics13111006)
Supplement: Supplementary file 1 [file antibiotics-13-01006-s001.zip › antibiotics-3237139 - Supplementary 2.pdf]

Table S1: The result in Table 1 shows a significant difference ($p<0.05$).

**Phage**	**Mean $\pm$ S. D**
Phage 10^3	$156.67^{b} \pm 50.33$
Phage10^6	96.00 ± 5.29
Phage 10^9	$0.00^{d} \pm 0.00$
Antibiotics alone	$103.33^{c} \pm 5.77$
Bacteria (control)	$300.00^{a} \pm 0.00$
Phage 10^3 + antibiotics	$103.33^{c} \pm 15.28$
Phage 10^6 + antibiotics	$1.33^{d} \pm 1.53$
Phage 10^9 + antibiotics	$0.00^{d} \pm 0.00$
**S. E$\pm$**	**10.860**
p-value	***0.000***

Values (in the same column) with the same subscript letters do not differ significantly from each other according to the Duncan multiple range test. **SE = Standard Error**

APPENDIX I

Source	Sum of Squares	df	Mean Square	F	Sig.
phage	218373.167	7	31196.167	88.177	.000
Error	5660.667	16	353.792		
Total	224033.833	23			

a. R Squared = .975 (Adjusted R Squared = .964)
