# Supplementary material for: Isolation and Characterization of a Novel Jumbo Phage HPP-Temi Infecting Pseudomonas aeruginosa Pa9 and Increasing Host Sensitivity to Ciprofloxacin
Source: Antibiotics (Basel). 2024 Oct 25;13(11):1006. doi: 10.3390/antibiotics13111006 (PMC11591403; doi:10.3390/antibiotics13111006)
Supplement: Supplementary file 1 [file antibiotics-13-01006-s001.zip › antibiotics-3237139 - Supplementary Figure S1.pdf]

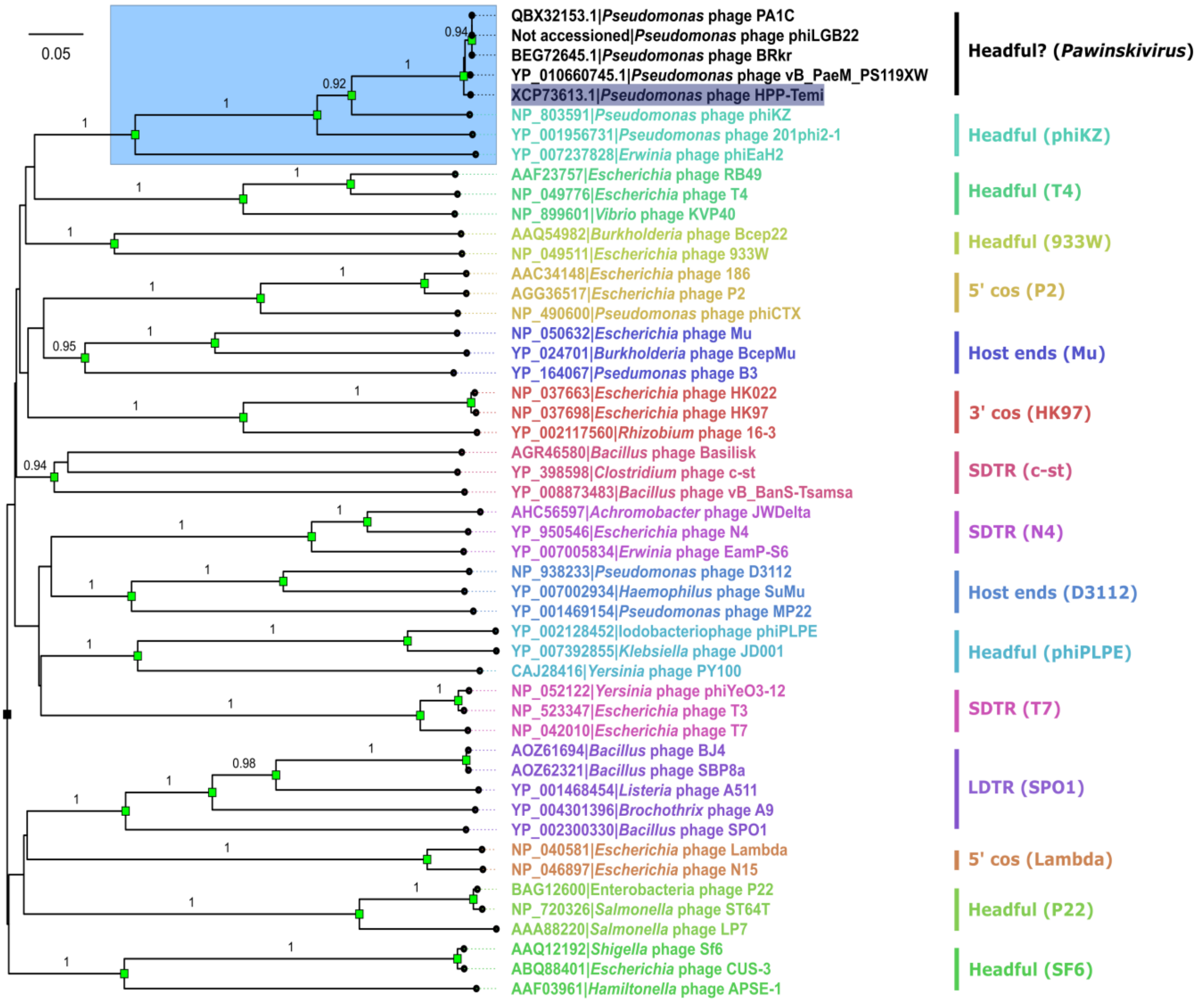

Figure S1. Neighbor-joining tree of terminase/terminase large subunit (TerL) amino acid sequences for the prediction of the tentative genome packaging strategy *Pawinskivirus* representatives use. Tip labels are colored based on the distinct packaging strategies the phages seen in the tree use (experimentally verified for most of the phages represented in the tree [39]). The fraction of replicate trees in which the associated sequences clustered together in the bootstrap test (1000 replicates) is shown next above the branches for branches having bootstrap support higher than 0.8 (such branches also have their distal nodes colored in green). The tree is drawn to scale, and branch lengths represent the number of amino acid substitutions per site. Tip labels correspond to the phages from which the respective terminase/TerL amino acid sequences were derived and are in the format of "Protein accession|Phage". Colored bars next to the labels indicate evolutionary dis-tinct TerL clades, which correspond to different packaging strategies (LDTR stands for long direct terminal repeats, SDTR—short direct terminal repeats). The analysis involved 50 amino acid se-quences. All multiple sequence alignment positions with less than 90% site coverage were elimi-nated. That is, fewer than 10% alignment gaps, missing data, and ambiguous bases were allowed at any position. There was a total of 267 positions in the final data set used to generate the tree.
